# Robust Lateral Stabilization Control of In-Wheel-Motor-Driven Mobile Robots via Active Disturbance Suppression Approach

**DOI:** 10.3390/s20185238

**Published:** 2020-09-14

**Authors:** Jie Meng, Shuting Wang, Liquan Jiang, Yuanlong Xie, Shiqi Zheng, Hao Wu

**Affiliations:** 1 School of Mechanical Science and Engineering, Huazhong University of Science and Technology, 1037 Luoyu Road, Wuhan 430074, China; mengjie_10@hust.edu.cn (J.M.); wangst@hust.edu.cn (S.W.); hustlqj@hust.edu.cn (L.J.); M201770409@hust.edu.cn (H.W.); 2 School of Automation, China University of Geosciences, 388 Lumo Road, Wuhan 430074, China; zhengshiqi@cug.edu.cn

**Keywords:** lateral stabilization control, mobile robots, active disturbance suppression, super-twisting sliding mode

## Abstract

Due to the praiseworthy maneuverability and actuation flexibility, the in-wheel-motor-driven mobile robots (IWMD-MR) are widely employed in various industrial fields. However, the active estimation and rejection of unknown disturbances/uncertainties remain a tough work for formulating a stable lateral motion controller. To address the challenge, this paper proposes a robust lateral stabilization control (RLSC) scheme for the developed IWMD-MR by designing an active disturbance suppression mechanism. The distinctive features of the proposed RLSC method are threefold: (i) With a fuzzy estimator, a modified super-twisting sliding mode method is designed to eliminate the system perturbations and time-varying lumped disturbances in an active manner; (ii) The resultant system trajectory is forced into a bounded switching region within finite time, which can be maintained therein for subsequent periods; (iii) Employing the Lyapunov function, new adaption rules for multivariable gains are derived to preserve the lateral motion stability and robustness. Finally, under the direct yaw moment control framework, simulation experiments of real-life IWMD-MR are offered to verify the effectiveness of the presented RLSC method.

## 1. Introduction

Since robotic technologies continue to develop in the automotive industry, mobile robots are being increasingly employed as a practical solution for mobile processing of large complex parts or logistics transportation [1,2,3,4]. Up to now, various types of mobile robots have been designed, such as two/three-wheeled mobile robots [5,6], differential mobile robots [7,8,9] and omnidirectional mobile robots equipped with Omni or Mecanum wheels [10,11]. Among the existing prototype, each wheel of two/three-wheeled or differential mobile robot usually has only one degree-of-freedom, implying the difficulties to perform omnidirectional movement flexibly in narrow spaces or confined environments [12]. As a typical differential-type mobile robot, the four-wheeled skid steering one can change its orientation by utilizing the actuation difference of each wheel, which may lead to inherent inefficiencies due to the skidding wheels [13]. Using Omni or Mecanum wheels, the omnidirectional mobile robot can realize effective omnidirectional movement without changing the direction of the mounted wheels. Such omnidirectional mobile robots allow for the lateral movement, but the rotation of rollers will result in the slippage of the mobile robot and the loss of ground friction interaction [14]. Moreover, the slippage is difficult to be modeled and identified. It is worth noting that in rigorous environments, the independently driven and steering architecture of four-wheeled autonomous mobile robots are beneficial for enhancing the maneuverability and applicability [15,16]. Specifically, the car-like in-wheel-motor-driven mobile robots (IWMD-MR) can sustain the free orientation regulation of the four-wheeled actuation system while providing better tolerance to surface irregularity and higher durability of tires. Given this context, IWMD-MR can achieve satisfying efficiency and actuation flexibility thanks to the special structure of the drivetrain [17,18,19].

Recently, the vigorous development of IWMD-MR enables immense technological and scientific advancements, particularly for lateral motion control [20,21]. With in-wheel or hub motors, the driving and braking torques on each wheel of IWMD-MR can be regulated more precisely. This actuation feature fosters lateral motion control superiority and can reach enhanced closed-loop stability and tracking control performance [22]. However, the lateral motion stabilization of IWMD-MR is more complicated due to the nonlinear dynamics, strong system coupling or interconnected states. Till now, it is still challenging for the IWMD-MR to achieve better controllability and stability under unknown disturbances/uncertainties such as modeling uncertainties, external disturbances (including the time-varying inertia and torque ripple) and parameter variations [23]. According to the different actuation characteristics, previous contributions mostly concentrate on the following two control methods:

(1) Active front steering control (AFSC). Receiving considerable interest from the mobile robotic fields, the potential benefits of the AFSC include improving handling the behavior during normal driving and guaranteeing lateral motion stability by utilizing the front steering commands. For example, in the presence of actuator faults including loss-of-effectiveness fault, additive fault and stuck-at-fixed-level fault, the motion stabilization issue of electric vehicles via AFSC is addressed in [24]; Based on the lateral tire force, Nam et al., present a robust AFSC method to strengthen the vehicle stability and maneuverability [25]; To optimize the front steering angle of autonomous vehicles, a model predictive control method is designed to trace the desired reference trajectories [26]. In [27,28], to realize active front steering of steer-by-wire systems, enhanced yaw stability controllers are designed with the verified effectiveness implemented on hardware-in-the-loop platforms. Even though the existing AFSC scheme can handle some of the lateral motion control issues, it is only applicable to control the considered IWMD-MR at a moderate cornering level.
(2) Direct yaw control (DYC). In addition to AFSC, the chassis DYC of the FAMR is able to achieve accurate yaw moment adjustment, leading to enhanced tracking performance with respect to dynamic tracking and asymptotical stability [29,30]. Different DYC related works have been explored, focusing on direct yaw moment scheduling of IWMD-MR. For seeking the optimal yaw moment and active steering angle, a multiobjective model predictive control method is provided to allocate the four-wheel torques and ensure the closed-loop stability of electrical vehicles [18]. By utilizing a robust control framework, Hu et al., resolve the motion stabilization issue of four-wheel electric vehicles to mitigate the modeling uncertainties, external disturbances and parameter variations [31]. To achieve yaw moment distribution, a hierarchical strategy is proposed by integrating an overlook controller and a servo-loop controller to effectively optimize the required yaw moment inputs of the IWMD-MR [32].

It should be pointed out that AFSC reaches additional steering by regulating the sideslip angle and producing lateral forces while DYC is capable of compensating the restrictions of the steering input. By utilizing different braking forces to the left and right sides of the wheels, DYC realizes the yaw motion using the steering angle information. However, the IWMD-MR system often suffers from uncertainness and various disturbances in practice, which imposes difficulties to achieve a dynamical lateral stabilization controller among the aforementioned studies. With superior advantages of insensitivity to system uncertainties, quick tracking response and easy implementation, sliding mode control (SMC) has been incorporated into the direct yaw moment control of mobile robotic systems over recent years. For instance, Ding et al., combine sliding mode mechanism and disturbance observer technology to explore the optimal yaw moment adaption law [33]; With practical demonstrations, an integral SMC solution is designed to enable a torque-vectoring regulation scheme for electric vehicles [34]. Indeed, the promising SMC solutions are potential for enhancing the control capabilities from both the disturbance alleviation and asymptotical stability [35]. By ensuring the finite-time or asymptotical convergence, it is possible to drive the constructed sliding variable to the desired sliding manifold under known bounded disturbance or its derivative. Nevertheless, the upper limitation information may be generally unknown due to the complex uncertainties and disturbances of the over-actuated IWMD-MR system considered here. On the other hand, although the SMC gains can quickly and exactly accommodate the time-varying operating conditions, the uncertainties cannot be directly reflected in the control parameters. Given this context, the control gains of such a passive mechanism should be relatively high to offer enough robustness for the resulting system while ensuring the dynamic tracking performance [36]. This is referred to as the overestimation problem of the control gains, and it may lead to serious chattering phenomena. To overcome the adverse influence, it is still of great significance to explore a more robust lateral stabilization controller for the IWMD-MR to ensure the stability and dynamic performance under unknown disturbances and uncertainties.

Motivated by the above analysis, we will focus on the robust DYC of the developed IWMD-MR in this paper. An improved robust lateral stabilization control (RLSC) is explored here to handle the active disturbance estimation and suppression issue of the IWMD-MR. The proposed RLSC scheme can simultaneously optimize the yaw rate and sideslip angle during the lateral motion control framework with guaranteed system robustness and dynamic tracking precision. This method treats the system vibrations, dynamic perturbations and external disturbance as complex uncertainties. Then, an enhanced super-twisting like sliding mode control algorithm is designed to estimate the uncertainties with a fuzzy-based estimator and reject the effects using modified adaption law. Under the presented RLSC method, the traditional passive adaption law of robust lateral stability control is modified to achieve active disturbance suppression performance. Moreover, the designed sliding variables can be quickly converged to the desired sliding manifold and maintained on it thereafter. Comparative experiments are performed in real-world scenarios, validating the benefits and superior abilities of the presented RLSC scheme.

The rest of this paper is constructed as follows: Section 2 provides the system modeling and problem formulation. The proposed RLSC scheme is presented in Section 3, including the presented active disturbance suppression structure and the related stability and finite-time convergence aspects. Further, Section 4 and Section 5 offer simulation experimental validations and concluding remarks, respectively.

## 2. System Modeling and Problem Formulation

### 2.1. System Modeling

**Figure 1:**
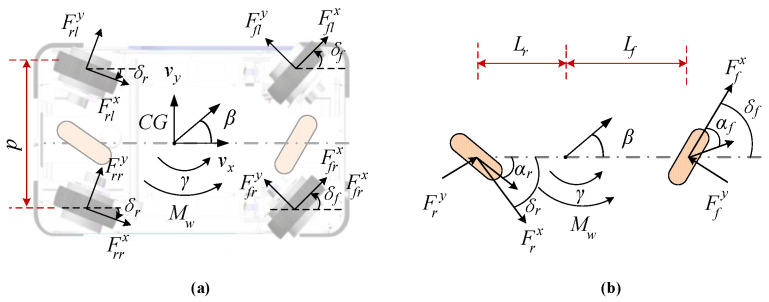
System modeling and problem formulation. (**a**) Four-wheeled model; (**b**) Single-track model.

For lateral motion control, the dynamic states of the considered IWMD-MR can be formulated as a multiple-input multiple-output system with interconnected variables, i.e., sideslip angle and yaw rate. For the two-degree-of-freedom planar model of the dynamical IWMD-MR system shown in Figure 1, we define the following notations: *m* total mass, *v_x_* longitudinal speed at the center of gravity (CG), $F_{i}^{x}$ and $F_{i}^{y}$ longitudinal and lateral tire forces at *i*th tire, respectively, *β* and *γ* sideslip angle and yaw rate, separately, *I_z_* inertia moment, *L_f_* and *L_r_* distances from the front and rear axles, respectively, *δ_f_* virtual front wheel angle, *δ_r_ = kδ_f_* virtual rear wheel angle with *k* being the user-defined coefficient that can be used for operating model configuration, *M_ω_* yaw moment generated by the traction moment of four wheels, *d* track width. In this paper, *k* is set as 0 to make the considered IWMD-MR operated in the widely adopted Ackerman mode.

In the yaw plane, the four-wheel expression of the IWMD-MR is determined by

(1) $$\left\{ \begin{array}{l} m\upsilon_{x}(\dot{\beta }+\gamma )=\sum_{i=1}^{2}\left(F_{i}^{x}\sin\delta_{f}+F_{i}^{y}\cos\delta_{f}\right)+\sum_{i=3}^{4}\left(F_{i}^{y}\cos\delta_{r}-F_{i}^{x}\sin\delta_{r}\right)\\ I_{z}\dot{\gamma }=\sum_{i=1}^{2}L_{f}(F_{i}^{x}\sin\delta_{f}+F_{i}^{y}\cos\delta_{f})-\sum_{i=3}^{4}L_{r}(F_{i}^{y}\cos\delta_{r}-F_{i}^{x}\sin\delta_{r})+M_{\omega }\\ M_{\omega }=0.5d(F_{rr}^{x}-F_{rl}^{x})\cos\delta_{r}+0.5d(F_{fr}^{x}-F_{fl}^{x})\cos\delta_{f} \end{array}\right.$$

In practice, we can directly measure or estimate the lateral forces $F_{rr}^{x},$ $F_{rl}^{x}$, $F_{fr}^{x}$ and $F_{fl}^{x}$ by using observer techniques [20]. As shown in Figure 1b, we will simplify the four-wheel model as a single-track model for the dynamic RLSC design. In this regard, we rewrite the lateral and yaw dynamics as follows

(2) $$\left\{ \begin{array}{l} m\upsilon_{x}(\dot{\beta }+\gamma )=F_{f}^{x}\cos\delta_{f}+F_{r}^{y}\cos\delta_{r}\\ I_{z}\dot{\gamma }=L_{f}F_{f}^{x}\cos\delta_{f}-L_{r}F_{r}^{y}\cos\delta_{r}+M_{\omega } \end{array}\right.$$

where the lateral tire forces $F_{f}^{x}$ and $F_{r}^{y}$ can be linearly calculated as follows

(3) $$F_{f}^{y}=-2C_{f}\left(\beta +\frac{L_{f}\gamma }{\upsilon_{x}}-\delta_{f}\right),\;F_{r}^{y}=-2C_{r}\left(\beta -\frac{L_{r}\gamma }{\upsilon_{x}}+\delta_{r}\right)$$

where $C_{f}$ and $C_{r}$ denote the cornering stiffnesses of the front and rear tires, separately.

In general, the tire concerning stiffness is affected by weight transfer. By employing small-angle approximation (e.g., $\cos\delta_{f}\approx 1$, $\cos\delta_{r}\approx 1$), we reconstruct the dynamic model in a general continuous-time form

(4) $$\dot{x}(t)=Ax(t)+Bu(t)+f(x,t)$$

where $x(t)={\left[\beta ,\gamma \right]}^{T}$ and $u(t)={\left[\delta_{f},M_{\omega }\right]}^{T}$ denote the state and control input vectors, separately, *f*(*x*,*t*) denotes the external disturbance, *A* and *B* denote the parametric vectors expressed by

$$A=\left[\begin{array}{cc} \frac{-2(C_{f}+C_{r})}{m\upsilon_{x}} & \frac{2(L_{r}C_{r}-L_{f}C_{f})}{m\upsilon_{x}^{2}}-1\\ \frac{-2(L_{f}C_{f}-L_{r}C_{r})}{I_{z}} & \frac{-2(L_{f}^{2}C_{f}+L_{r}^{2}C_{r})}{I_{z}\upsilon_{x}} \end{array}\right],\;B=\left[\begin{array}{cc} \frac{2C_{f}-2C_{r}k}{m\upsilon_{x}} & 0\\ \frac{2L_{f}C_{f}+2L_{r}C_{r}k}{I_{z}} & \frac{1}{I_{z}} \end{array}\right]$$

Then, considering the parametric perturbation, one can further obtain the IWMD-MR system determined by (4) as below

(5) $$\dot{x}(t)=(\overset{⏜}{A}+\Delta A)x(t)+(\overset{⏜}{B}+\Delta B)u(t)+f(x,t)$$

where $\overset{⏜}{A}$ and $\overset{⏜}{B}$ denote the nominal parametric matrix derived by using $C_{f}={\overset{⏜}{C}}_{f}$ and $C_{r}={\overset{⏜}{C}}_{r}$, respectively, ${\overset{⏜}{C}}_{f}$ and ${\overset{⏜}{C}}_{r}$ denote the related nominal parameters, Δ*A* and Δ*B* denote the uncertainties caused by the vibrations of the tire concerning stiffness.

Define the parametric perturbation *D*(*x*,*t*) as D(x,t)≜ΔAx(t)+ΔBu(t). By constructing the relationship between *D*(*x*,*t*) and *d*(*x*,*t*), we can always find a $\overset{⏜}{B}$ to satisfy (6). However, the solution process needs to calculate ${\overset{⏜}{B}}^{-1}$, so we have formulated the following assumptions for the subsequent derivations:

A1. The pair (*A*,*B*) is controllable;
A2. The vector *B* has full column rank and it is invertible in this paper.

Therefore, the following matching condition is satisfied

(6) $$D(x,t)=\overset{⏜}{B}d(x,t)$$

where *d*(*x*,*t*) denotes the parametric vector.

For a practical implementation, we will limit the system states and the optimized control input to some known bounds due to the mechanical characteristics. Based on (6), the bounds of *d*(*x*,*t*) and *d*(*x*,*t*) can be derived accordingly

(7) $$d(x,t)={\overset{⏜}{B}}^{-1}(\Delta Ax(t)+\Delta Bu(t)),\;\dot{d}(x,t)={\overset{⏜}{B}}^{-1}(\Delta A\dot{x}(t)+\Delta B\dot{u}(t))$$

Denote the lumped disturbances as $f^{\prime }(x,t)=d(x,t)+f(x,t)$. With full consideration of (6) and (7), it is concluded that *f*′(*x*,*t*) and the related derivative *f*′(*x*,*t*) are limited by respective known positive constants *d_m_* and ${\tilde{d}}_{m}$. That is to say,

(8) $$\left\|f^{\prime }(x,t)\right\|<d_{m},\;\left\|\dot{f^{\prime }}(x,t)\right\|<{\tilde{d}}_{m}$$

Hence, the considered system can be finally constructed by

(9) $$\dot{x}(t)=\overset{⏜}{A}x(t)+\overset{⏜}{B}u(t)+f^{\prime }(x,t)$$

Further, we offer the following assumption:

A3. The function *f*′(*x*,*t*) and the related gradient are bounded by unknown functions.

**Remark 1.**  *It is well known that the mentioned system uncertainties and disturbances are bounded, as demonstrated in (8). On the other hand, due to the time-varying characteristics, we cannot directly obtain the vibration information of the lumped disturbances. Given this context, an enhanced super-twisting like sliding mode control algorithm is designed to estimate the lumped disturbances with a fuzzy-based estimator and then mitigate the influences using the modified adaption law in this paper.*

### 2.2. Problem Formulation

As shown in Equations (5) and (9), on has two system states to be controlled and two controllable inputs, i.e., *δ_f_* and *M_ω_*. In practice, the yaw rate and sideslip angle can be regarded as the commonly applied indicators of lateral stability and dynamic performance. Furthermore, the required yaw rate *γ_d_* is attained based on the desired trajectories. Specifically, we will use a reference mode to derive the IWMD-MR based on the monitored information, such as the steering angle and longitudinal velocity *v_x_*. In this paper, the concerning *γ_d_* and *β_d_* of the vehicle are derived as follows

(10) $$\gamma_{d}=\min\{|\tilde{\gamma }|,|\gamma_{\max}|\}\cdot sign(\delta_{f})$$

(11) $$\beta_{d}=\min\{|\tilde{\beta }|,|\beta_{\max}|\}\cdot sign(\delta_{f})$$

where *γ_max_* and *β_max_* denote the practical bounds of the desired signals, and $\tilde{\gamma }$ and $\tilde{\beta }$ is determined by

(12) $$\tilde{\gamma }=\frac{\upsilon_{x}}{dK_{s}}\delta_{f}$$

(13) $$\tilde{\beta }=\frac{L_{r}2dL_{r}C_{r}-L_{r}mL_{f}\upsilon_{x}^{2}}{2dL_{r}C_{r}dK_{s}}\delta_{f}$$

(14) $$K_{s}=1+\frac{m\left(L_{r}C_{r}-L_{f}C_{f}\right)\upsilon_{x}^{2}}{2d^{2}C_{f}C_{r}}$$

Therefore, the trajectory-tracking problem considered in this work is translated into the direct yaw moment control issue of the IWMD-MR to stabilize the lateral motion activities relating to yaw moment and sideslip angle. To this end, this paper will propose an RLSC control scheme with active disturbance suppression capability for the disturbed IWMD-MR system, realizing the lateral stability control when following the desired profiles. We will explore an adaptive-gain-scheduled algorithm to optimize the continuous control inputs in the presence of unknown additive and multiplicative disturbances and perturbations.

## 3. Main Results

### 3.1. The Proposed Control Structure

Based on the above-referenced assumptions (A1–A3), using the linear state transformation

(15) $$\left(\begin{array}{c} \varphi_{1}\\ \varphi_{2} \end{array}\right)=Tx,\;T=\left[\begin{array}{c} \vec{B}\\ \overset{\leftarrow }{B} \end{array}\right],\;\overset{\leftarrow }{B}B=0,\;\vec{B}={\left(B^{T}B\right)}^{-1}B^{T}$$

one can rewrite the system (9) as follows

(16) $$\left\{ \begin{array}{l} {\dot{\varphi }}_{1}(t)={\tilde{A}}_{11}\varphi_{1}(t)+{\tilde{A}}_{12}\varphi_{2}(t)\\ {\dot{\varphi }}_{2}(t)={\tilde{A}}_{21}\varphi_{1}(t)+{\tilde{A}}_{22}\varphi_{2}(t)+u(t)+g(\varphi_{1},\varphi_{2},t) \end{array}\right.$$

where ${\tilde{A}}_{11}\in {\mathbb{R}}^{\left(n_{1}-n_{2})\times (n_{1}-n_{2}\right)},\;{\tilde{A}}_{12}\in {\mathbb{R}}^{(n_{1}-n_{2})\times n_{2}},{\tilde{A}}_{21}\in {\mathbb{R}}^{n_{2}\times (n_{1}-n_{2})},\;{\tilde{A}}_{22}\in {\mathbb{R}}^{n_{2}\times n_{2}}$ with *n*_1_ and *n*_2_ being the dimensional of the input vector and output vector, respectively, and *g*(*φ*_1_,*φ*_2_,*t*) denotes the complex uncertainties that cannot be directly measured.

Using a designed matrix $G\in {\mathbb{R}}^{n_{2}\times (n_{1}-n_{2})}$ which can be derived utilizing linear control methods (e.g., eigenvalue assignment), the desired sliding mode surface of this paper is constructed as

(17) $$s=\varphi_{2}-G\varphi_{1}$$

Then, the control input takes the form of

(18) $$u=-\left({\tilde{A}}_{21}+{\tilde{A}}_{22}G-G({\tilde{A}}_{11}+{\tilde{A}}_{12}G)\right)\varphi_{1}(t)-\left({\tilde{A}}_{22}-G{\tilde{A}}_{12}\right)s+v$$

where *v* denotes the enforcement control law to be designed later.

Therefore, the system (16) takes the form

(19) $$\left\{ \begin{array}{l} {\dot{\varphi }}_{1}=\left({\tilde{A}}_{11}+{\tilde{A}}_{12}G\right)\varphi_{1}+{\tilde{A}}_{12}s\\ \dot{s}=v+g(\varphi_{1},\varphi_{2},t) \end{array}\right.$$

In addition, with $\phi_{1}(s)=\mu_{1}|s{|}^{\alpha }sign(s)+\mu_{2}s$, where *μ_i_*_=1,2_ > 0 denote predefined coefficients, the sliding mode reaching conditions can be satisfied by applying the following enforcement law

(20) $$v=-\sigma_{1}\phi_{1}(s)-\sigma_{2}\phi_{2}(s)$$

(21) $$\begin{array}{cc} {\dot{\phi }}_{2}(s) & ={\dot{\phi }}_{1}(s)\phi_{1}(s)\\ & =\mu_{1}^{2}\alpha |s{|}^{2\alpha -1}sign(s)+(1+\alpha )\mu_{1}\mu_{2}|s{|}^{\alpha }sign(s)+\mu_{2}^{2}s \end{array}$$

where *σ*_1_ and *σ*_2_ denote the adaptive gains to be derived later, α∈(0,1) is the specified fractional order.

Subsisting (20) into (19), we obtain

(22) $$\dot{s}=-\sigma_{1}\left(\mu_{1}|s{|}^{\alpha }sign(s)+\mu_{2}s\right)+\upsilon +g(\varphi_{1},\varphi_{2},t)$$

(23) $$\dot{\upsilon }=-\sigma_{2}\left(\mu_{1}^{2}\alpha |s{|}^{2\alpha -1}sign(s)+(1+\alpha )\mu_{1}\mu_{2}|s{|}^{\alpha }sign(s)+\mu_{2}^{2}s\right)$$

Since the complex uncertainty cannot be directly measured or calculated, an adaptive fuzzy-based estimator is designed here to approximate the unknown disturbances/uncertainties, so as to achieve active suppression ability and avoid the overestimation of the related control gains. We construct a fuzzy-based approximation $\hat{g}(x|\theta_{g})={\hat{\theta }}_{g}^{T}\lambda (x)$ where ${\hat{\theta }}_{g}$ denotes the adaptive law based on the Lyapunov stability theory and *λ*(*x*) denotes the fuzzy basis vector. A series of fuzzy rules are used to estimate the uncertainties $g(x|\theta_{g})$:

Rule (i): IF *x*_1_ is $A_{1}^{l_{1}}$ and … *x_n_* is $A_{n}^{l_{n}}$, THEN $\hat{g}$ is $E^{l_{1},\ldots ,l_{n}}$

Where related fuzzy sets $A_{i}^{l_{j}}$, *x_i_* are input variables, *i* = 1,2,…,*n*.

Then, by employing the traditional center average defuzzifier, we can calculate the estimator’s output as

(24) $$\hat{g}(x|{\hat{\theta }}_{g})=\frac{\sum_{l_{1}=1}^{p_{1}}\cdots \sum_{l_{n}=1}^{p_{n}}{\bar{y}}_{g}^{l_{1},\ldots ,l_{n}}\left(\prod_{i=1}^{n}\mu_{A_{i}^{l_{j}}}(x_{i})\right)}{\sum_{l_{1}=1}^{p_{1}}\cdots \sum_{l_{n}=1}^{p_{n}}\left(\prod_{i=1}^{n}\mu_{A_{i}^{l_{j}}}(x_{i})\right)}$$

where $\mu_{A_{i}^{l_{j}}}(x_{i})$ denotes the membership function.

Suppose that a $\prod_{i=1}^{n}p_{i}$-dimensional fuzzy basis vector is defined as *λ*(*x*) with input variable *x*. Thus, we can rewrite (24) as

(25) $$\hat{g}(x|\theta_{g})={\hat{\theta }}_{g}^{T}\lambda (x),\;\lambda_{l_{1},\ldots ,l_{n}}(x)=\frac{\prod_{i=1}^{n}\mu_{A_{i}^{l_{j}}}(x_{i})}{\sum_{l_{1}=1}^{p_{1}}\cdots \sum_{l_{n}=1}^{p_{n}}\left(\prod_{i=1}^{n}\mu_{A_{i}^{l_{j}}}(x_{i})\right)}$$

The optimal parameter vector is noted as $\theta_{g}^{*T}$ and one can obtain

(26) $$g^{*}(x|\theta_{g}^{*})=\underset{\theta_{g}\in \Omega_{\varepsilon }}{\arg\min}\left\{\sup\left|\hat{g}(x|\theta_{g})-\varepsilon_{g}\right|\right\}\lambda (x)$$

where *ε_g_* denotes the approximated error and Ω*_θ_* is the set of *θ_g_*.

Defining ${\tilde{\theta }}_{\gamma }=\theta_{\gamma }^{*}-{\hat{\theta }}_{\gamma }$, one can be derived that

(27) $$\hat{g}(x|\theta_{g})=\theta_{g}^{*T}\lambda (x)+\varepsilon_{g}-{\hat{\theta }}_{g}^{T}\lambda (x)={\tilde{\theta }}_{g}^{T}\lambda (x)+\varepsilon_{g}$$

### 3.2. Adaption Law Design

**Theorem** **1.**  *Under the proposed control law derived by (18), (22) and (23) and the fuzzy-based estimator (25), there exist a range of adaptive values σ_i=1,2_ such that s and* $\dot{s}$ *can be driven to the origin in finite time and maintained on it thereafter in any initial condition. To be more specific, the resultant system can achieve finite-time convergence as soon as the condition*

(28) $$\sigma_{1}\geq \delta +\frac{1}{2\rho }\left\{2\rho +3\varepsilon +\frac{2\rho \hat{g}(x|\theta_{g})}{\phi_{1}^{\prime }\phi_{1}}\right\}$$

*holds and if the following adaption laws are employed*

(29) $${\dot{\sigma }}_{1}=\{\begin{array}{cc} \varpi_{1}\sqrt{\omega_{1}/2} & if\;\sigma_{1}>\sigma_{1}^{m}\\ \eta & if\;\sigma_{1}\leq \sigma_{1}^{m} \end{array}$$

(30) $$\sigma_{2}=\frac{1}{\varepsilon }\left\{\sigma_{1}\varepsilon +\rho +\varepsilon -\varepsilon \frac{\hat{g}(x|\theta_{g})}{\phi_{1}^{\prime }\phi_{1}}\right\}$$

*where ρ > 0, ε > 0, δ > 0,* $\sigma_{1}^{m}>0$ *and η ≥ 0 denote arbitrary constants.*

**Proof.**  The proof can be classified into three parts.

Part 1: the existence of the fuzzy-based estimation law and the adaption gain $\sigma_{i=1,2}(s,\dot{s},t)$.

With the help of (22) and (23), we choose a Lyapunov function *V*_1_(*s*,*v*) for the subsystem of $\varsigma^{T}={\left(\phi_{1}(s),\upsilon \right)}^{T}$ using a quadratic form

(31) $$V_{1}(s,\upsilon )=\frac{1}{2}\varsigma^{T}P\varsigma$$

where *P* = *P*^T^ > 0 denotes a symmetric matrix with elements to be determined later, i.e.,

(32) $$P=\left[\begin{array}{cc} P_{1} & P_{3}\\ P_{3} & P_{2} \end{array}\right]$$

The consideration of (22) leads to

(33) $$\dot{\varsigma }={\phi^{\prime }}_{1}\left[\begin{array}{cc} -\sigma_{1} & 1\\ -\sigma_{2} & 0 \end{array}\right]\varsigma +\left[\begin{array}{c} g(x|\theta_{g})\\ 0 \end{array}\right]$$

where ${\phi^{\prime }}_{1}=\frac{\partial \phi_{1}}{\partial s}$ denotes a positive scalar.

Assuming that there exists a function J(t) that results in $\frac{g(x|\theta_{g})}{{\phi^{\prime }}_{1}}\frac{1}{J(t)}=\phi_{1}$, we obtain that

(34) $$\dot{\varsigma }={\phi^{\prime }}_{1}\left[\begin{array}{cc} -\sigma_{1}+J(t) & 1\\ -\sigma_{2} & 0 \end{array}\right]\varsigma$$

Substituting $\Lambda ≜\left[\begin{array}{cc} -\sigma_{1}+J(t) & 1\\ -\sigma_{2} & 0 \end{array}\right]$ into (34) yields

(35) $$\dot{\varsigma }={\phi^{\prime }}_{1}\Lambda \varsigma$$

Besides, one can obtain that

(36) $$\begin{array}{cc} \Lambda^{T}P+P\Lambda & =[\begin{array}{cc} -\sigma_{1}+J(t) & -\sigma_{2}\\ 1 & 0 \end{array}][\begin{array}{ll} P_{1} & P_{3}\\ P_{3} & P_{2} \end{array}]+[\begin{array}{ll} P_{1} & P_{3}\\ P_{3} & P_{2} \end{array}][\begin{array}{cc} -\sigma_{1}+J(t) & 1\\ -\sigma_{2} & 0 \end{array}]\\ & =\left[\begin{array}{cc} 2\left(-\sigma_{1}+J(t)\right)P_{1}-2\sigma_{2}P_{3} & \left(-\sigma_{1}+J(t)\right)P_{3}-\sigma_{2}P_{2}+P_{1}\\ \left(-\sigma_{1}+J(t)\right)P_{3}-\sigma_{2}P_{2}+P_{1} & 2P_{3} \end{array}\right] \end{array}$$

According to (36), design a symmetric matrix *Q* as

(37) $$\begin{array}{cc} Q & =-\Lambda^{T}P-P\Lambda \\ & =\left[\begin{array}{cc} 2\sigma_{2}P_{3}-2\left(-\sigma_{1}+J(t)\right)P_{1} & \sigma_{2}P_{2}-P_{1}-\left(J(t)-\sigma_{1}\right)P_{3}\\ \sigma_{2}P_{2}-P_{1}-\left(J(t)-\sigma_{1}\right)P_{3} & -2P_{3} \end{array}\right] \end{array}$$

Then, choosing a positive definite $P=\left[\begin{array}{cc} \rho +\varepsilon & -\varepsilon \\ -\varepsilon & \varepsilon \end{array}\right]=\left[\begin{array}{ll} P_{1} & P_{3}\\ P_{3} & P_{2} \end{array}\right]$ (ρ,ε>0) results in

(38) $$\begin{array}{cc} Q & =\left[\begin{array}{cc} -2\sigma_{2}\varepsilon -2\left(-\sigma_{1}+J(t)\right)\left(\rho +\varepsilon \right) & -\left(\rho +\varepsilon \right)+\left(J(t)-\sigma_{1}\right)\varepsilon +\sigma_{2}\varepsilon \\ -\left(\rho +\varepsilon \right)+\left(J(t)-\sigma_{1}\right)\varepsilon +\sigma_{2}\varepsilon & 2\varepsilon \end{array}\right]\\ & =\left[\begin{array}{cc} -2\sigma_{2}\varepsilon +2\sigma_{1}\rho +2\sigma_{1}\varepsilon -2J(t)\rho -2J(t)\varepsilon & \sigma_{2}\varepsilon -\sigma_{1}\varepsilon +J(t)\varepsilon -\rho -\varepsilon \\ \sigma_{2}\varepsilon -\sigma_{1}\varepsilon +J(t)\varepsilon -\rho -\varepsilon & 2\varepsilon \end{array}\right] \end{array}$$

With the universal Approximation Theorem [37] and the fuzzy-based estimator, we can determine the uncertainties in J(t) with

(39) $$\hat{J}(t)=\frac{\hat{g}(x|{\hat{\theta }}_{g})}{{\phi^{\prime }}_{1}\phi_{1}}$$

If the control gain *σ*_2_ is designed as

(40) $$\sigma_{2}=\sigma_{1}-J(t)+\frac{\rho }{\varepsilon }+1$$

we can reformulate Equation (38) with

(41) $$Q=\left[\begin{array}{cc} \underset{\Phi }{\underbrace{2\sigma_{1}\rho -2\rho -2\varepsilon }}-2\hat{J}(t)\rho & 0\\ 0 & 2\varepsilon \end{array}\right]=\left[\begin{array}{cc} q_{11} & q_{12}\\ q_{21} & q_{22} \end{array}\right]$$

Further, the derivative of *V*_1_(*s*,*v*) is determined by

(42) $$\begin{array}{cc} {\dot{V}}_{1}(s,\upsilon ) & ={\dot{\varsigma }}^{T}P\varsigma +\varsigma^{T}P\dot{\varsigma }\\ & =-{\phi^{\prime }}_{1}\varsigma^{T}Q\varsigma \\ & =-{\phi^{\prime }}_{1}(q_{11}\phi_{1}^{2}+q_{22}\upsilon^{2})\\ & =2\rho \tilde{g}\phi_{1}-2\rho g\phi_{1}-{\phi^{\prime }}_{1}\Phi \phi_{1}^{2}-{\phi^{\prime }}_{1}q_{22}\upsilon^{2} \end{array}$$

where $\tilde{g}(t)=\hat{g}(t)-g(t)$.

By constructing $V_{2}({\tilde{\theta }}_{\gamma })=\frac{1}{2\varpi }{\tilde{\theta }}_{g}^{T}{\tilde{\theta }}_{g}$ and ${\dot{\tilde{\theta }}}_{g}={\dot{\theta }}_{g}^{*}-{\dot{\hat{\theta }}}_{g}=-{\dot{\hat{\theta }}}_{g}$, where ϖ denotes a positive constant, it is concluded that ${\dot{V}}_{2}({\tilde{\theta }}_{g})=-\frac{1}{\varpi }{\tilde{\theta }}_{g}^{T}{\dot{\hat{\theta }}}_{g}$ and

(43) $$\begin{array}{cc} {\dot{V}}_{1}(s,\upsilon )+{\dot{V}}_{2}({\tilde{\theta }}_{g}) & =2\rho \tilde{g}\phi_{1}-2\rho g\phi_{1}-{\phi^{\prime }}_{1}\Phi \phi_{1}^{2}-{\phi^{\prime }}_{1}q_{22}\upsilon_{2}-\frac{1}{\varpi }{\tilde{\theta }}_{g}^{T}{\dot{\hat{\theta }}}_{g}\\ & ={\tilde{\theta }}_{g}^{T}\left(2\rho \lambda (x)\phi_{1}-\frac{1}{\varpi }{\dot{\hat{\theta }}}_{g}\right)-2\rho g\phi_{1}-{\phi^{\prime }}_{1}\Phi \phi_{1}^{2}-{\phi^{\prime }}_{1}q_{22}\upsilon_{2}+\varepsilon_{g} \end{array}$$

Therefore, the learning law of the fuzzy-based estimator is then derived, i.e.,

(44) $${\dot{\hat{\theta }}}_{g}=2\rho \lambda (x)\phi_{1}\varpi$$

The combination of (43) and (44) results in

(45) $$\begin{array}{cc} {\dot{V}}_{1}(s,\upsilon )+{\dot{V}}_{2}({\tilde{\theta }}_{g}) & =-2\rho g\phi_{1}-{\phi^{\prime }}_{1}\Phi \phi_{1}^{2}-{\phi^{\prime }}_{1}q_{22}\upsilon^{2}+\varepsilon_{g}\\ & =-{\phi^{\prime }}_{1}[2\rho J+\Phi ]\phi_{1}^{2}-{\phi^{\prime }}_{1}q_{22}\upsilon^{2}+\varepsilon_{g}\\ & =-{\phi^{\prime }}_{1}\varsigma^{T}\tilde{Q}\varsigma +\varepsilon_{g} \end{array}$$

where $\tilde{Q}=\left[\begin{array}{cc} \frac{2\rho g(x|\theta_{g})}{\phi_{1}^{\prime }\phi_{1}}+2\sigma_{1}\rho -2\rho -2\varepsilon & 0\\ 0 & 2\varepsilon \end{array}\right]$.

Since the approximation error *ε_g_* is sufficiently small, it is easy to choose a positive *ε* that satisfies $\varepsilon \geq \varepsilon_{g}+C$, where C is an arbitrarily small positive constant. Thus, we conclude that

(46) $$\tilde{Q}-\left[\begin{array}{cc} \varepsilon & 0\\ 0 & \varepsilon \end{array}\right]=\left[\begin{array}{cc} \frac{2\rho g(x|\theta_{g})}{\phi_{1}^{\prime }\phi_{1}}+2\sigma_{1}\rho -2\rho -3\varepsilon & 0\\ 0 & \varepsilon \end{array}\right]$$

To ensure $\tilde{Q}-diag(\varepsilon ,\varepsilon )\geq 0$, the consideration of Algebraic Riccati Inequality [38] leads to

(47) $$\frac{2\rho g(x|\theta_{g})}{\phi_{1}^{\prime }\phi_{1}}+2\sigma_{1}\rho -2\rho -3\varepsilon \geq 0,\;\varepsilon \geq 0$$

Therefore, based on (28), we conclude that (47) can be guaranteed. In this way, the matrix $\tilde{Q}$ is positive definite and its minimal eigenvalue satisfies $\lambda_{\min}(\tilde{Q})$ satisfies $\lambda_{\min}(\tilde{Q})\geq \varepsilon$.

Part 2: we will focus on the analysis of finite-time convergence.

Defining $V(\varsigma ,{\tilde{\theta }}_{g})≜V_{1}(s,\upsilon )+V_{2}({\tilde{\theta }}_{g})$, the magnitude of $\dot{V}(\varsigma ,{\tilde{\theta }}_{g})$ can be further minified as

(48) $$\begin{array}{cc} \dot{V}(\varsigma ,{\tilde{\theta }}_{g}) & \leq -{\phi^{\prime }}_{1}\varsigma^{T}\tilde{Q}\varsigma \\ & =-\varepsilon \left(\frac{\alpha \mu_{1}+\mu_{2}{\left|s\right|}^{1-\alpha }}{{\left|s\right|}^{1-\alpha }}\right)\varsigma^{T}\varsigma \leq -\varepsilon \left(\alpha \mu_{1}\mu_{2}\frac{1}{2\mu_{2}{\left|s\right|}^{1-\alpha }}+\frac{\mu_{2}}{4}\right){\left\|\varsigma \right\|}_{2}^{2} \end{array}$$

where ${\left\|\varsigma \right\|}_{2}^{2}=\phi_{1}^{2}+\upsilon_{2}=\mu_{1}^{2}{\left|s\right|}^{2\alpha }+2\mu_{1}\mu_{2}{\left|s\right|}^{\alpha +1}+\mu_{2}^{2}s^{2}+\upsilon^{2}$ is the Euclidean norm of *ζ*.

Since $V(\varsigma ,{\tilde{\theta }}_{g})=\frac{1}{2}\varsigma^{T}P\varsigma +\frac{1}{2\varpi }{\tilde{\theta }}_{g}^{T}{\tilde{\theta }}_{g}$ and 1−α∈(0,1), defining $\frac{1}{\varpi }{\left\|{\tilde{\theta }}_{g}\right\|}_{2}^{2}=\Theta (t){\left\|\varsigma \right\|}_{2}^{2}$, we can get the following inequalities

(49) $$\mu_{2}{\left|s\right|}^{1-\alpha }\leq {\left\|\varsigma \right\|}_{2}\leq \frac{2V^{1/2}(\varsigma ,{\tilde{\theta }}_{g})}{{\left[\lambda_{\min}\{P\}+\Theta \right]}^{1/2}},\;{\left\|\varsigma \right\|}_{2}\geq \frac{2V^{1/2}(\varsigma ,{\tilde{\theta }}_{g})}{{\left[\lambda_{\max}\{P\}+\Theta \right]}^{1/2}}$$

Based on (49), it is possible to write (48) as

(50) $$\begin{array}{cc} \dot{V}(\varsigma ,{\tilde{\theta }}_{g}) & \leq -\varepsilon \left(\alpha \mu_{1}\mu_{2}\frac{1}{2\mu_{2}{\left|s\right|}^{1-\alpha }}+\mu_{2}\right){\left\|\varsigma \right\|}_{2}^{2}\\ & \leq -\frac{\alpha \varepsilon \mu_{1}\mu_{2}{\left[\lambda_{\min}\{P\}+\Theta \right]}^{1/2}}{\left[\lambda_{\max}\{P\}+\Theta \right]}V^{1/2}(\varsigma ,{\tilde{\theta }}_{g})-\frac{\varepsilon \mu_{2}}{\lambda_{\max}\{P\}+\Theta }V(\varsigma ,{\tilde{\theta }}_{g}) \end{array}$$

which demonstrates that $\dot{V}(\varsigma ,{\tilde{\theta }}_{g})$ is negative definite and the closed-loop stability of the resultant system can be guaranteed.

Denoting $\gamma_{1}(\mu_{1},\mu_{2})≜\frac{\alpha \varepsilon \mu_{1}\mu_{2}{\left[\lambda_{\min}\{P\}+\Theta \right]}^{1/2}}{\left[\lambda_{\max}^{}\{P\}+\Theta \right]}$ $\gamma_{2}(\mu_{2})≜\frac{\varepsilon \mu_{2}}{\lambda_{\max}\{P\}+\Theta }$, since the solution of the differential equation

(51) $$\dot{v}=-\gamma_{1}(\mu_{1},\mu_{2})v^{1/2}-\gamma_{2}(\mu_{2})v,v(0)=v_{0}>0$$

can be provided with

(52) $$v(t)=\exp(-\gamma_{2}(\mu_{2})t){\left[v_{0}^{1/2}+\frac{\gamma_{1}(\mu_{1},\mu_{2})}{\gamma_{2}(\mu_{2})}\left(1-\exp\left(\frac{\gamma_{2}(\mu_{2})}{2}t\right)\right)\right]}^{2}$$

it is derived that *V*(*t*) ≤ *v*(*t*) when *V*(*s_0_*,*v_0_*) ≤ *v*_0_.

Especially, the reaching time of Equation (52) can be computed by

(53) $$T=\frac{2}{\gamma_{2}(\mu_{2})}\ln\left(\frac{\gamma_{2}(\mu_{2})}{\gamma_{1}(\mu_{1},\mu_{2})}V^{1/2}(s_{0},\upsilon_{0})+1\right)$$

Therefore, under the designed variable gains determined by Equation (40), *s* and $\dot{s}$ can be converged to the origin in finite time, and the reaching time can be estimated by Equation (53).

Part 3: Analysis of the adaptive control gains.

Introduce the following Lyapunov function $\tilde{V}(\varsigma ,{\tilde{\theta }}_{g},\sigma_{1},\sigma_{2})$

(54) $$\tilde{V}(\varsigma ,{\tilde{\theta }}_{g},\sigma_{1},\sigma_{2})=V(\varsigma ,{\tilde{\theta }}_{g})+\frac{1}{2\omega_{1}}{\left(\sigma_{1}-\sigma_{1}^{*}\right)}^{2}+\frac{1}{2\omega_{2}}{\left(\sigma_{2}-\sigma_{2}^{*}\right)}^{2}$$

where $\sigma_{1}^{*}>0$, $\sigma_{2}^{*}>0$ denote the optimal control gains, *ω*_2_ > 0 denotes a positive constant.

With $\varepsilon_{\sigma_{1}}≜\sigma_{1}-\sigma_{1}^{*}$ and $\varepsilon_{\sigma_{2}}≜\sigma_{2}-\sigma_{2}^{*}$, the derivative of $\tilde{V}(\varsigma ,{\tilde{\theta }}_{g},\sigma_{1},\sigma_{2})$ is given by

(55) $$\begin{array}{c} \dot{\tilde{V}}(\varsigma ,{\tilde{\theta }}_{g},\sigma_{1},\sigma_{2})\\ =\dot{V}(\varsigma ,{\tilde{\theta }}_{g})+\frac{1}{\omega_{1}}\varepsilon_{\sigma_{1}}{\dot{\sigma }}_{1}+\frac{1}{\omega_{2}}\varepsilon_{\sigma_{2}}{\dot{\sigma }}_{2}\\ \leq -\gamma_{1}V_{1/2}(\varsigma ,{\tilde{\theta }}_{g})+\frac{1}{\omega_{1}}\varepsilon_{\sigma_{1}}{\dot{\sigma }}_{1}+\frac{1}{\omega_{2}}\varepsilon_{\sigma_{2}}{\dot{\sigma }}_{2}\\ =-\gamma_{1}V_{1/2}(\varsigma ,{\tilde{\theta }}_{g})-\frac{\varpi_{1}}{\sqrt{2\omega_{1}}}\left|\varepsilon_{\sigma_{1}}\right|-\frac{\varpi_{2}}{\sqrt{2\omega_{2}}}\left|\varepsilon_{\sigma_{2}}\right|+\frac{1}{\omega_{1}}\varepsilon_{\sigma_{2}}{\dot{\sigma }}_{1}+\frac{1}{\omega_{2}}\varepsilon_{\sigma_{2}}{\dot{\sigma }}_{2}+\frac{\varpi_{1}}{\sqrt{2\omega_{1}}}\left|\varepsilon_{\sigma_{1}}\right|+\frac{\varpi_{2}}{\sqrt{2\omega_{2}}}\left|\varepsilon_{\sigma_{2}}\right| \end{array}$$

where $\varpi_{2}>0$ denotes a positive constant.

The integration of ${\left(x^{2}+y^{2}+z^{2}\right)}^{1/2}\leq \left|x\right|+\left|y\right|+\left|z\right|$ and (55) yields

(56) $$-\gamma_{1}V_{1/2}(\varsigma ,{\tilde{\theta }}_{g})-\frac{\varpi_{1}}{\sqrt{2\omega_{1}}}\left|\varepsilon_{\sigma_{1}}\right|-\frac{\varpi_{2}}{\sqrt{2\omega_{2}}}\left|\varepsilon_{\sigma_{2}}\right|\leq -\min(\gamma_{1},\varpi_{1},\varpi_{2})\sqrt{\tilde{V}(\varsigma ,{\tilde{\theta }}_{\gamma },\sigma_{1},\sigma_{2})}$$

Using $\eta_{0}=\min(\gamma_{1},\varpi_{1},\varpi_{2})$, we can rewrite (55) as

(57) $$\dot{\tilde{V}}(\varsigma ,{\tilde{\theta }}_{g},\sigma_{1},\sigma_{2})\leq -\eta_{0}\sqrt{\tilde{V}(\varsigma ,{\tilde{\theta }}_{g},\sigma_{1},\sigma_{2})}+\frac{1}{\omega_{1}}\varepsilon_{\sigma_{1}}{\dot{\sigma }}_{1}+\frac{1}{\omega_{2}}\varepsilon_{\sigma_{2}}{\dot{\sigma }}_{2}+\frac{\varpi_{1}}{\sqrt{2\omega_{1}}}\left|\varepsilon_{\sigma_{1}}\right|+\frac{\varpi_{2}}{\sqrt{2\omega_{2}}}\left|\varepsilon_{\sigma_{2}}\right|$$

Since there exist a positive constant $\sigma_{1}^{*}>0$, $\sigma_{2}^{*}>0$ satisfying $\sigma_{1}-\sigma_{1}^{*}<0$ and $\sigma_{2}-\sigma_{2}^{*}<0$, we can reduce (57) as

(58) $$\dot{\tilde{V}}(\varsigma ,{\tilde{\theta }}_{g},\sigma_{1},\sigma_{2})\leq -\eta_{0}\sqrt{\tilde{V}(\varsigma ,{\tilde{\theta }}_{g},\sigma_{1},\sigma_{2})}-\left\{\left|\varepsilon_{\sigma_{1}}\right|\left(\frac{1}{\omega_{1}}{\dot{\sigma }}_{1}-\frac{\varpi_{1}}{\sqrt{2\omega_{1}}}\right)+\left|\varepsilon_{\sigma_{2}}\right|\left(\frac{1}{\omega_{2}}{\dot{\sigma }}_{2}-\frac{\varpi_{2}}{\sqrt{2\omega_{2}}}\right)\right\}$$

For the case $\sigma_{1}\geq \sigma_{1}^{m}$ for all the t>0, by choosing the adaption of the gains

(59) $${\dot{\sigma }}_{1}=\varpi_{1}\sqrt{\frac{\omega_{1}}{2}},\;{\dot{\sigma }}_{2}=\varpi_{2}\sqrt{\frac{\omega_{2}}{2}}$$

we can guarantee that

(60) $$\dot{\tilde{V}}(\varsigma ,{\tilde{\theta }}_{g},\sigma_{1},\sigma_{2})\leq -\eta_{0}\sqrt{\tilde{V}(\varsigma ,{\tilde{\theta }}_{g},\sigma_{1},\sigma_{2})}$$

Based on the Lyapunov theory, we can conclude that the resultant closed-loop system can achieve strict finite-time convergence.

For the case $\sigma_{1}<\sigma_{1}^{m}$, we known *σ*_1_ will increase immediately as $\sigma_{1}=\sigma_{1}+\eta t$. Then, as soon as $\sigma_{1}$ becomes greater or equal to $\sigma_{1}^{m}$, the condition that defines the case $\sigma_{1}\geq \sigma_{1}^{m}$ holds in finite time.

Further, one can select suitable parameters to guarantee that (59) and (30) coincide. Therefore, under the proposed adaption control gains, *s* and $\dot{s}$ can be forced to the origin in finite time, and the system states will be converged on the desired sliding manifold. This completes the proof. □

**Remark 2.**  *It should be noted that for the motion control of a system subject to uncertainties or disturbances, the control gain should be configurated to offer enough robustness for the controlled system. This may cause the overestimation issue of the control gains and the undesired chattering phenomenon. In this paper, by using the additional fuzzy-based estimator to approximate and compensate the disturbances in a feedforward way, the overestimation of the control gains can be well addressed intuitively. In this regard, the dynamic tracking and system robustness can be enhanced simultaneously.*

## 4. Simulation Experimental Validations

### 4.1. Experimental Implementation

**Figure 2:**
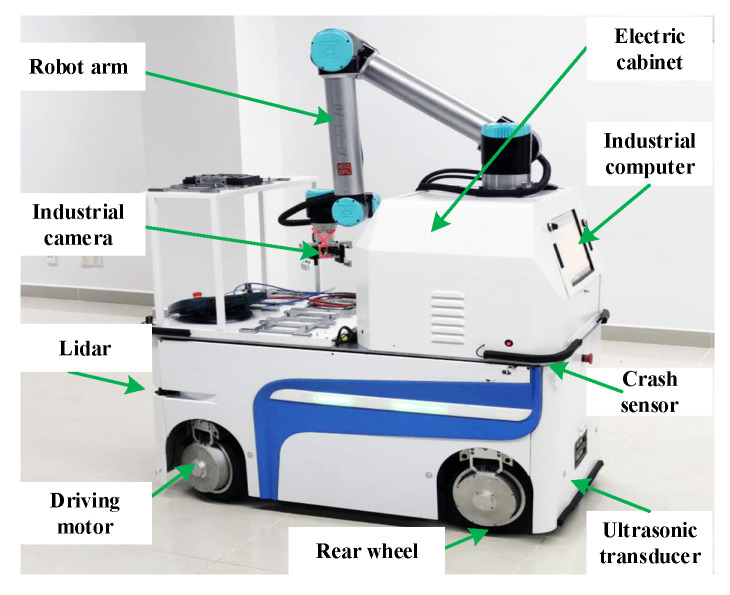
The prototype of the developed IWMD-MR.

**Figure 3:**
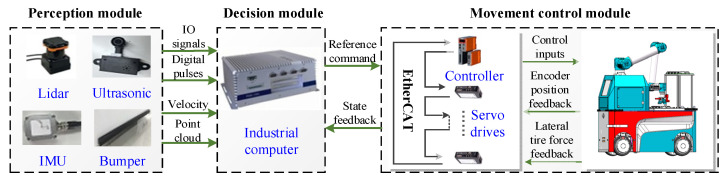
The construction of hardware architecture.

**Figure 4:**
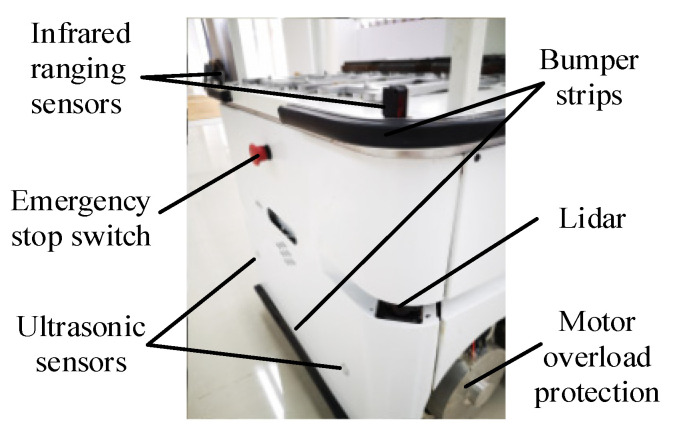
Multi-sensor safety module.

To verify the proposed RLSC scheme, the developed IWMD-MR is considered for simulation validation, as shown in Figure 2. For onboard equipment, the developed IWMD-MR is equipped with an electric cabinet, industrial computer, lidar (HOKUYO UTM-30LX, Hokuyo Automatic CO., LTD, Osaka, Japan), ultrasonic transducer, crash sensor, industrial camera and robot arm. It has been applied to industrial manufacturing applications to synchronously perform locomotion and manipulation. This IWMD-MR has several outstanding characteristics, including automatic charging, trackless autonomous navigation, obstacle avoidance detection and vision-based workpiece operation. Owing to the independently driving independently steering property, each wheel can achieve active arbitrary movements and rotation, realizing a lateral stabilization control scheme. As can be seen from Figure 3, the hardware architecture contains the modules of: (1) perception, used to obtain the sensory date for perceiving the real-world surrounding and guaranteeing the safety of the robot in unmapped or dynamic environments; (2) decision making, used for global and local path planning and formulating strategies for next actions; (3) movement control, implementing the actuation functions to realize yaw moment control. Specifically, the sideslip angle can be estimated using lateral tire force sensors [20]. Then, according to the point cloud data of the lidar, the IMU measurement including magnetic field strength from the magnetometer, acceleration from the accelerometer and angular velocity from the gyroscope, and the displacement feedback obtained by the encoder, the robot real-time pose and accurate yaw rate can be obtained using multi-sensor fusion [39,40]. At this point, the external observation data yaw rate and sideslip angle can be acquired. In addition, as shown in Figure 4, the IWMD-MR adds a multi-sensor safety module to ensure the safety of the navigation system.

For comparison simulation experiments, according to the features of the developed IWMD-MR platform, the related controller gains and parameters are determined by: *l_f_* = *l_r_* = 0.48 m, *d* = 0.53 m, *m* = 700 kg, *μ*_1_ = 0.2, *μ*_2_ = 0.7, $\varpi_{1}=1$, *ω*_1_ = 2, *ε* = 0.002, *ρ* = 1, *δ* = 0.01 and *α* = 0.7. For a fair comparison, we use the following control schemes for comparison: (1) traditional proportional-integral-derivative (PID) controller tuned by trial and error with *k_p_* = 0.3, *k_i_* = *k_d_* = 1.2; (2) the proposed super-twisting SMC method without fuzzy-based estimator; (3) the proposed RLSC method with the presented fuzzy-based estimator for active disturbance suppression. The sampling time is specified as 0.001 s. Moreover, for the fuzzy-based estimator, the following memberships are applied:

(61) $$1/\exp\left[5*\mathrm{sign}\left(x\right)\left(x-a_{1}\right)/b_{1}^{2}\right],\;\exp\left[-\frac{{\left(x-a_{2}\right)}^{2}}{b_{2}^{2}}\right],\;\exp\left[-\frac{{\left(x-a_{3}\right)}^{2}}{b_{3}^{2}}\right]\;\exp\left[-\frac{{\left(x-a_{4}\right)}^{2}}{b_{4}^{2}}\right],\exp\left[-\frac{{\left(x-a_{5}\right)}^{2}}{b_{5}^{2}}\right],1/\exp\left[5*\mathrm{sign}\left(x\right)\left(x-a_{6}\right)/b_{6}^{2}\right]$$

where for the yaw rate aspect, we select

(62) $$\left\{ \begin{array}{cc} a & =\left[\begin{array}{ccccc} a_{1} & a_{2} & a_{3} & a_{4} & \begin{array}{cc} a_{5} & a_{6} \end{array} \end{array}\right]\\ & =\left[\begin{array}{cccccc} 0.0325 & 0.0450 & 0.0825 & 0.12 & 0.1575 & 0.175 \end{array}\right]\\ b & =\left[\begin{array}{ccccc} b_{1} & b_{2} & b_{3} & b_{4} & \begin{array}{cc} b_{5} & b_{6} \end{array} \end{array}\right]\\ & =\left[\begin{array}{cccccc} 0.025 & 0.025 & 0.025 & 0.025 & 0.025 & 0.025 \end{array}\right] \end{array}\right.$$

while for the estimation of the uncertainties in the sideslip angle aspect, we choose

(63) $$\left\{ \begin{array}{l} a=\left[\begin{array}{cccccc} 0.0065 & 0.0113 & 0.0206 & 0.03 & 0.0394 & 0.044 \end{array}\right]\\ b=\left[\begin{array}{cccccc} 0.0125 & 0.0056 & 0.0056 & 0.0056 & 0.00565 & 0.0125 \end{array}\right] \end{array}\right.$$

### 4.2. Experimental Results and Discussions

**Figure 5:**
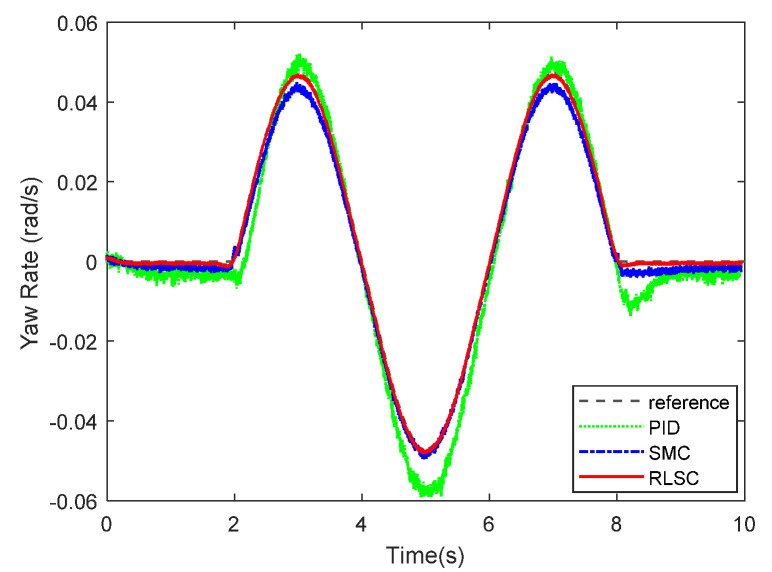
Yaw rate tracking response of Case 1.

**Figure 6:**
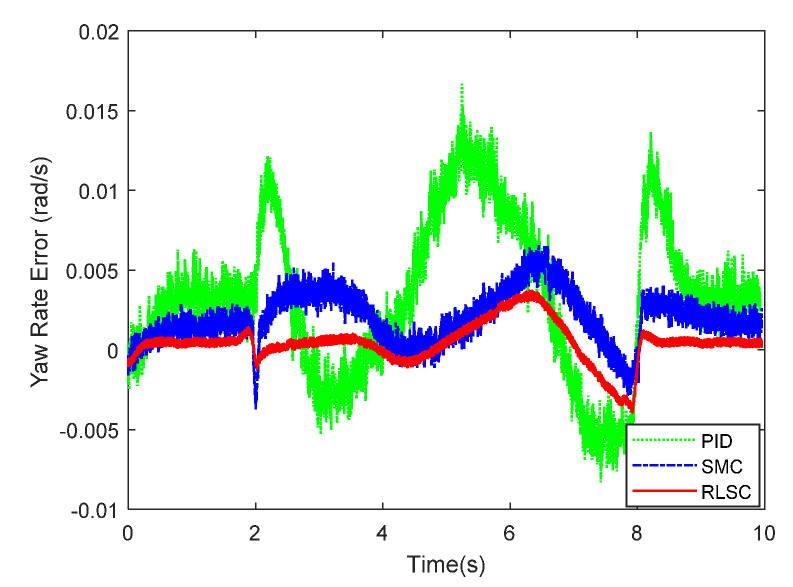
Yaw rate tracking errors of Case 1.

For real-world experimental validation, we will consider the single lane-changing maneuvers for the developed IWMD-MR system under the traditional widely used Ackerman mode. The maneuvering responses concerning the yaw rate and sideslip angle can be generally applied to evaluate the lateral motion control performance in nonidealized working conditions. Given this context, we provide the following cases for experimental validation.

(Case 1): In this case, the IWMD-MR is operated under a low ground interaction friction coefficient. A sinusoidal-like trajectory is considered here. The lateral motion tracking response of yaw rate and related errors are demonstrated in Figure 5 and Figure 6, separately. From these two figures, one can conclude that both the comparative approaches are able to stabilize the dynamic tracking errors. For the traditional PID controller, it may lead to huge overshoots when tracking the time-varying profiles. In comparison, with the sliding mode rules of the traditional SMC and our RLSC methods, the transient performances of the yaw rate tracking responses are apparently improved. To be more specific, the concerned responses with our RLSC has lower overshoots and steady-state errors than those with the standard PID or SMC method.

**Figure 7:**
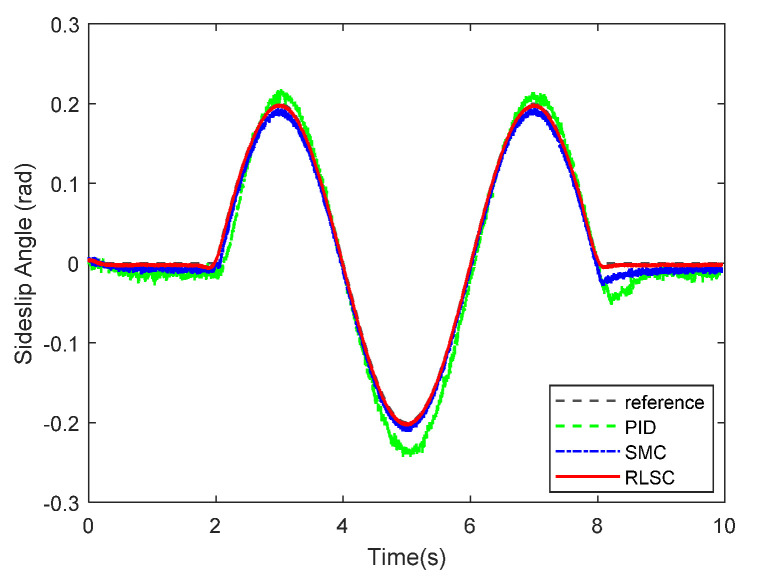
Sideslip angle tracking response of Case 1.

**Figure 8:**
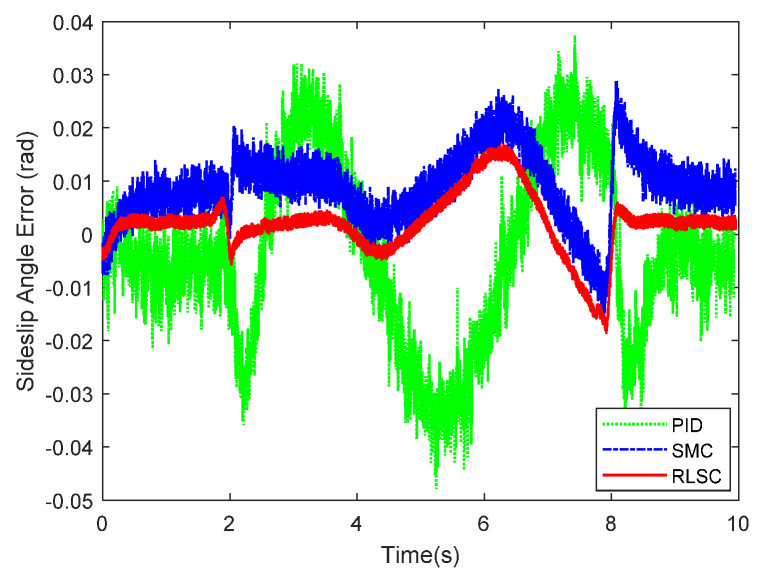
Sideslip angle tracking errors of Case 1.

Furthermore, the achieved results of the investigated sideslip angle and corresponding tracking errors are demonstrated in Figure 7 and Figure 8, respectively. By observing these results, both of the RLSC and SMC can maintain the tracking errors within smaller limits, but the proposed RLSC is able to enhance the transient performance relating to overshoot mitigation and error reduction. The vibration tendency of the system trajectory coincides with that of the desired curvature of the reference profiles. As can be seen from the yaw rate and sideslip angle tracking responses, due to the system disturbances and uncertainties, some fluctuations can be observed in the system states under both the traditional PID and SMC methods. By using the presented fuzzy-based estimator, the system perturbations and time-varying lumped disturbances can be accommodated in an active way. Therefore, the achieved lateral motion trajectories are smoother and steadier.

**Figure 9:**
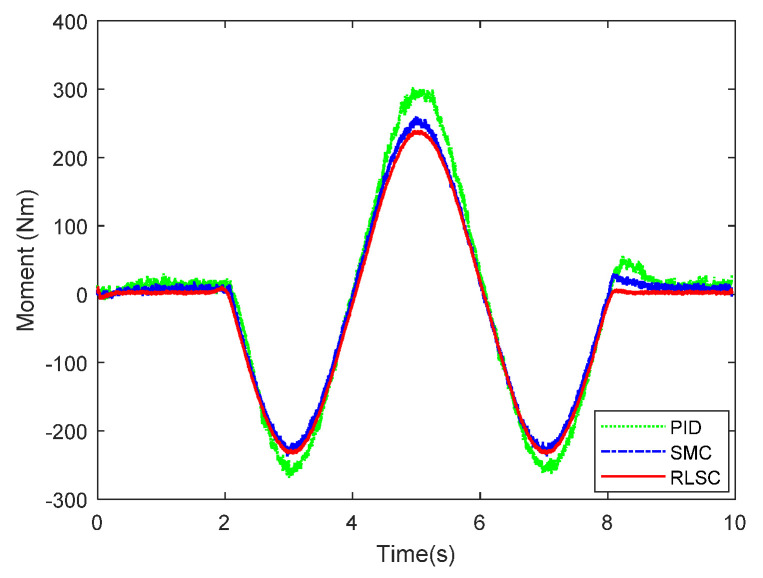
Direct yaw moment input of Case 1.

**Figure 10:**
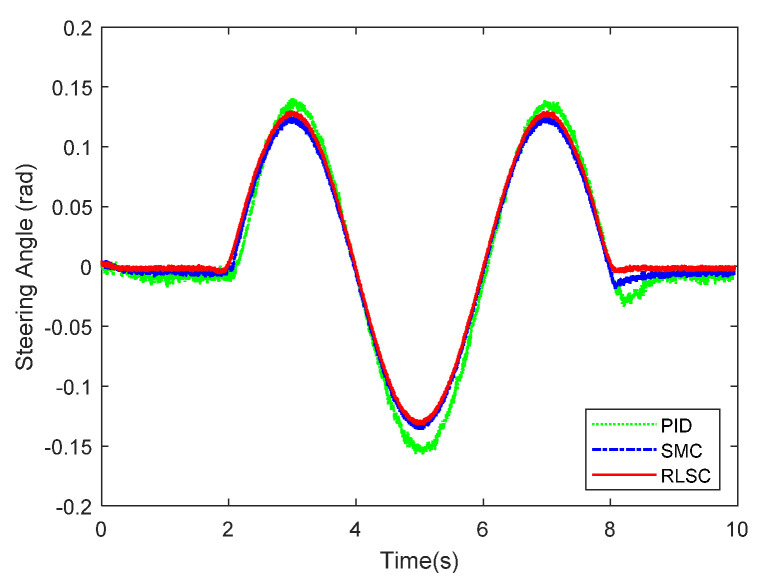
Front steering wheel angle of Case 1.

**Figure 11:**
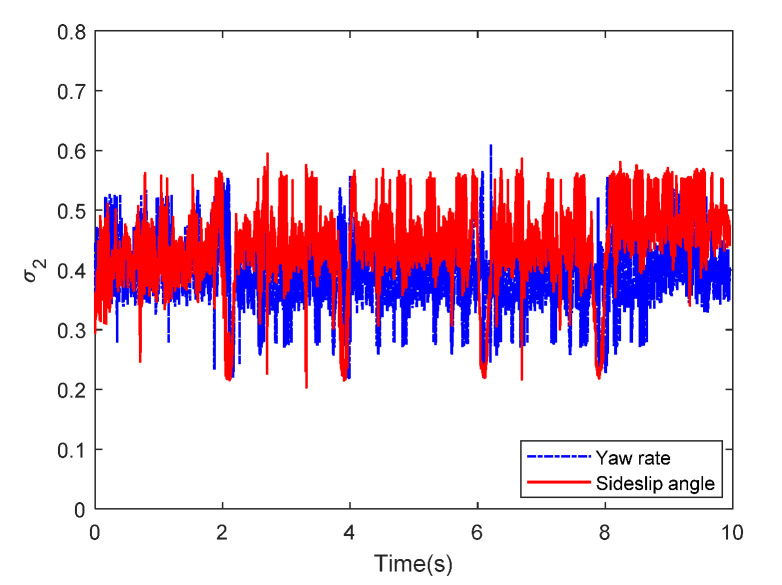
The related control gain $\sigma_{2}$ of Case 1.

**Figure 12:**
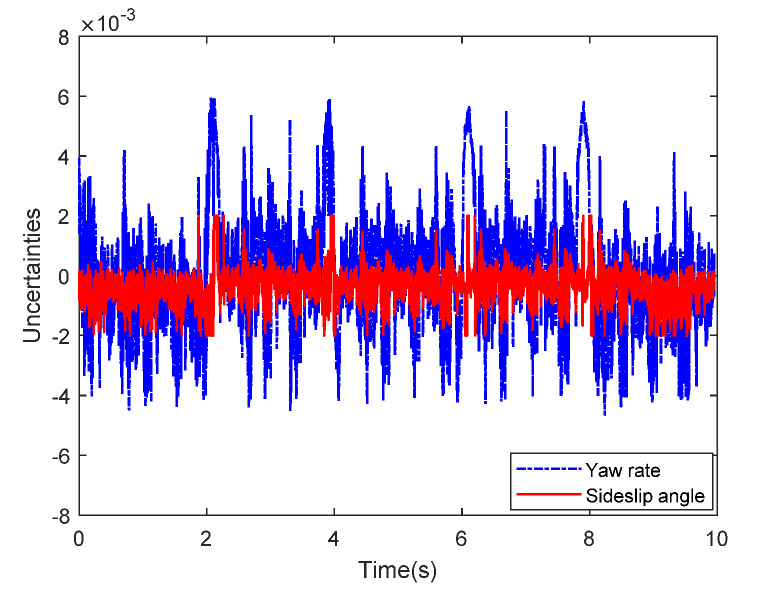
The estimated uncertainties of Case 1.

**Figure 13:**
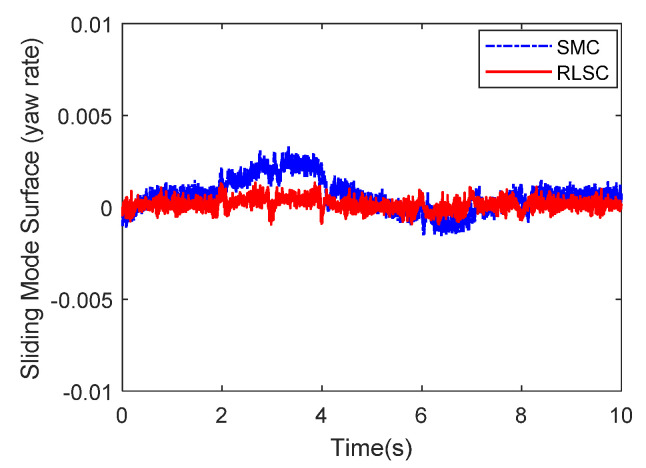
Sliding mode surface (yaw rate) of Case 1.

**Figure 14:**
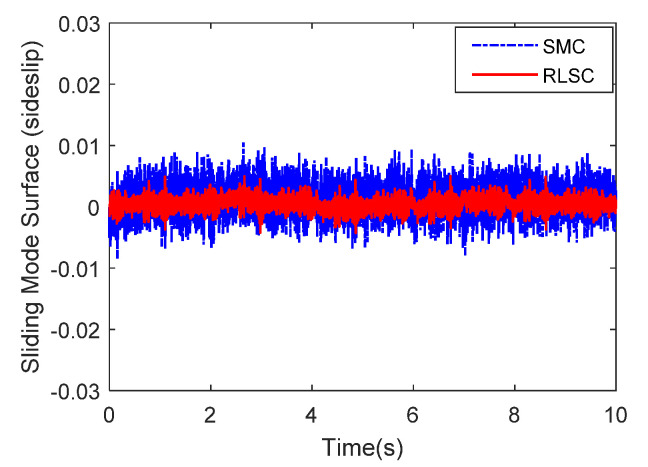
Sliding mode surface (sideslip angle) of Case 1.

Then, the concerning external yaw moment and front steering angle are exhibited in Figure 9 and Figure 10, separately. As shown in Figure 9 and Figure 10, the control inputs using the comparison methods are steadily optimized with reasonable magnitude. Similarly, the proposed RLSC is capable of reducing the input overshoots, leading to a smoother dynamic tracking response. Meanwhile, we have listed the vibrations of the control gain *σ*_2_ and the estimated uncertainties in Figure 11 and Figure 12, separately. As mentioned before, the method of using a fixed control gain is prone to overestimation problems in order to increase robustness. In contrast, thanks to the adaption laws, the control gain *σ*_2_ will dynamically be adjusted as the disturbance changes, and the lateral motion control performance has been improved significantly. At the same time, by taking the estimated uncertainties into the formulation of the control law, we can directly estimate and mitigate the undesired influence to achieve an active disturbance suppression framework, therefore enhancing the lateral stabilization motion control performance. Furthermore, we have presented the related sliding mode surfaces in Figure 13 and Figure 14. These results demonstrate that by using the SMC method, the sliding mode variables are within small regions around zero, and the proposed RLSC scheme can attain a smoother response as compared to the traditional SMC method.

From the above-mentioned analyses, the proposed RLSC method is capable of reducing the tracking overshoots, thus mitigating the tracking errors and increasing the path-following accuracy. It can be also concluded that the RLSC method maintains the control advantages of the sliding mode mechanism, and is much faster and more precise than the standard SMC method. This validates the practicability of the proposed RLSC for the controlled system subjects to perturbations and time-varying lumped disturbances.

(Case 2): To test the robustness of the resulting IWMD-MR system under the widely applied Ackerman mode, the trap cut steering profile is considered here. It should be mentioned that the IWMD-MR is operated with high ground interaction friction in this case.

**Figure 15:**
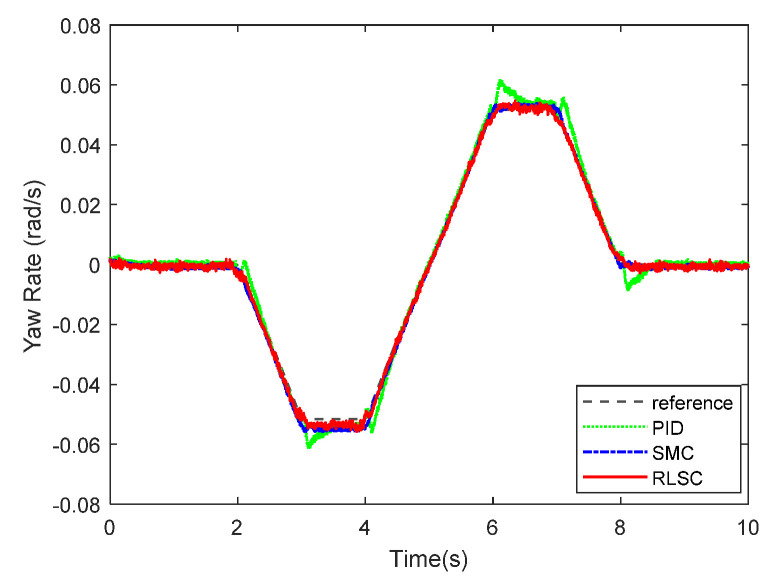
Yaw rate tracking response of Case 2.

**Figure 16:**
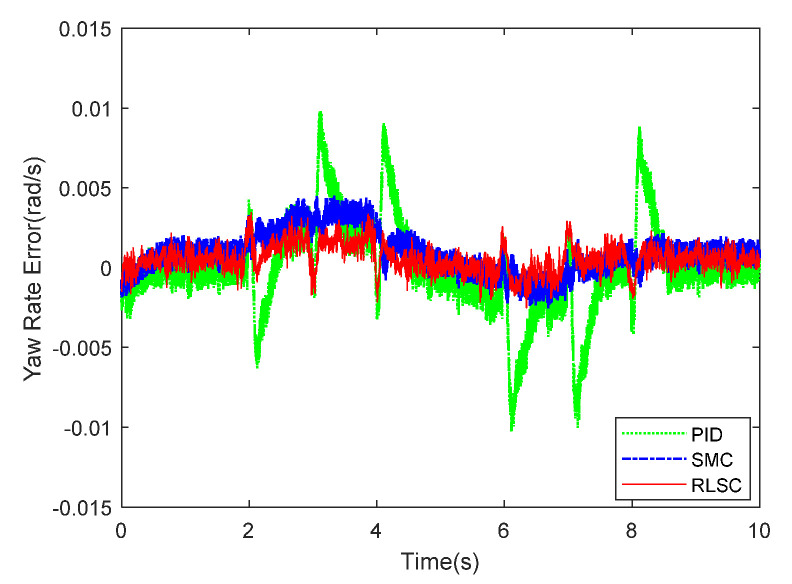
Yaw rate tracking errors of Case 2.

The experimental results of the yaw rate response exhibited in Figure 15 and Figure 16 show the corresponding tracking errors. From Figure 15 and Figure 16, one can conclude that the trajectory tracking errors of the yaw rate can be stabilized by the three comparison approaches. By applying the proposed RLSC, the lateral offset can be driven to steady states quickly, which is very critical for the autonomous mobile robots (including IWMD-MR) in emergency situations. Compared with the traditional methods (i.e., PID controller and SMC technique), RLSC can practically mitigate the response overshoot and increase the dynamic response. It should be pointed out that the tracking errors obtained here are relatively smaller than in Case 1. The reason lies in that the reference path curvature employed in Case 2 is much smaller than that in Case 1, and the steady-state errors of the yaw rate tracking can be reduced by our RLSC method during dynamic tracking.

**Figure 17:**
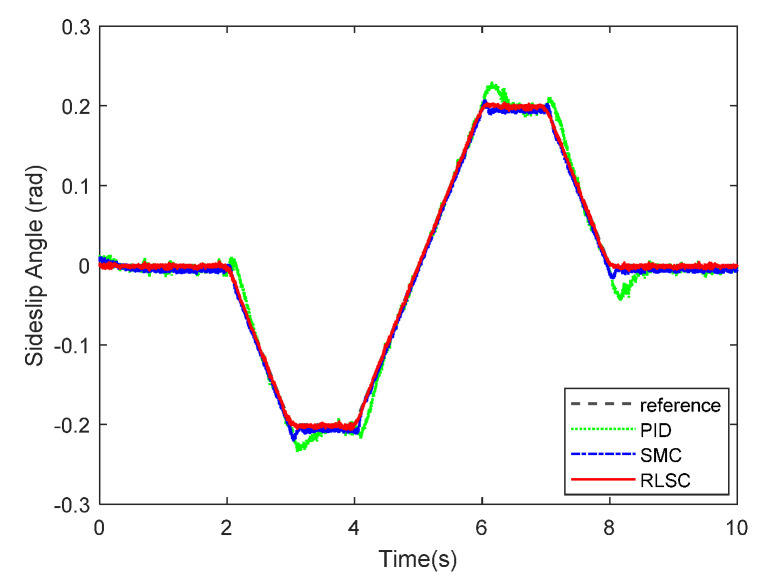
Sideslip angle tracking response of Case 2.

**Figure 18:**
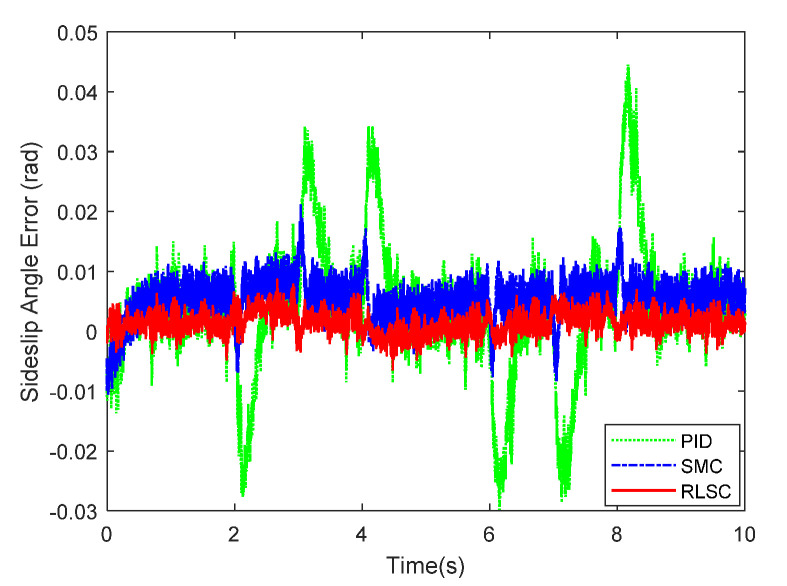
Sideslip angle tracking errors of Case 2.

**Figure 19:**
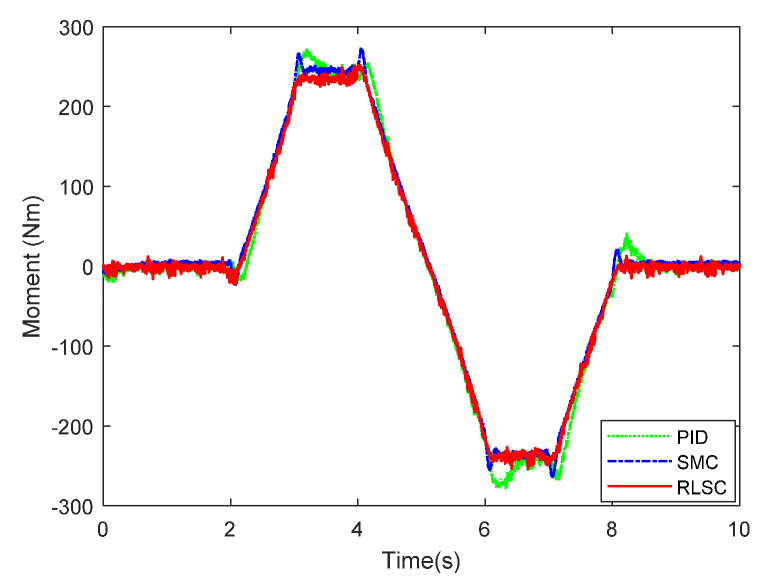
Direct yaw moment input of Case 2.

**Figure 20:**
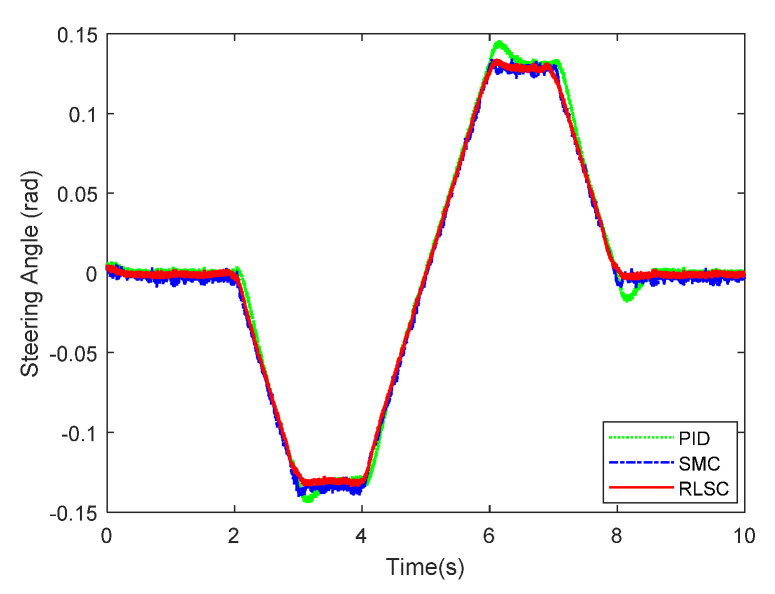
Front steering angle of Case 2.

**Figure 21:**
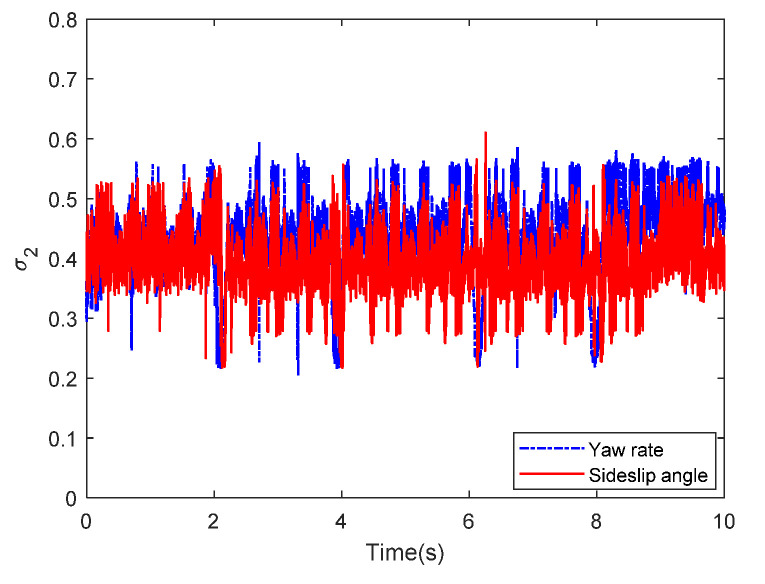
The related control gain $\sigma_{2}$ of Case 2.

**Figure 22:**
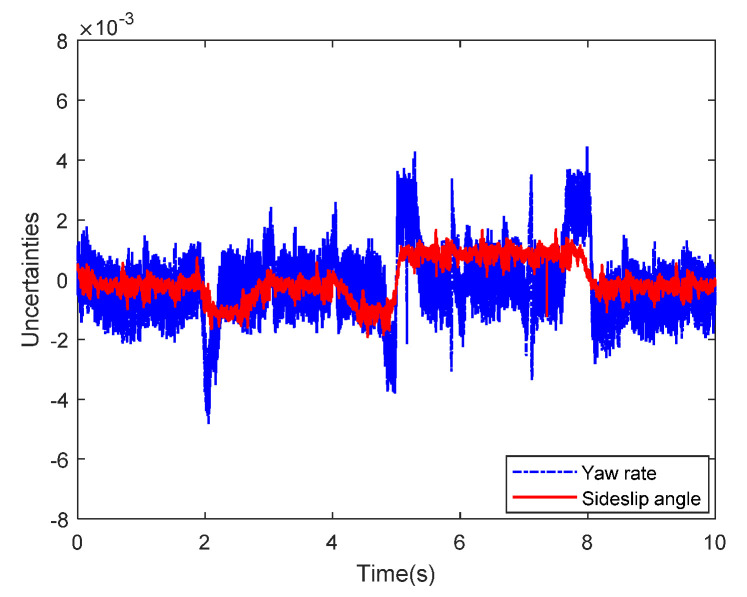
The estimated uncertainties of Case 2.

**Figure 23:**
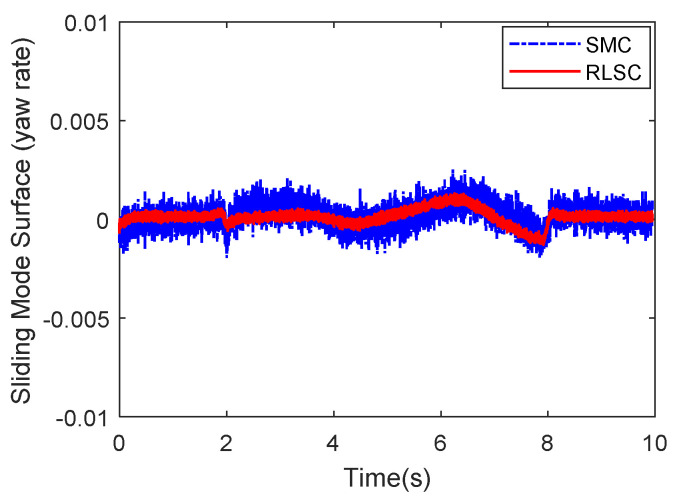
Sliding mode surface (yaw rate) of Case 2.

**Figure 24:**
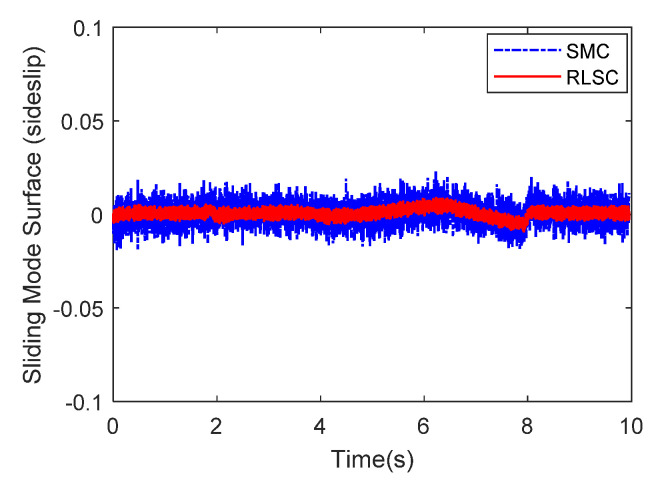
Sliding mode surface (sideslip angle) of Case 2.

Likewise, the sideslip angle tracking response and related errors are presented in Figure 17 and Figure 18, separately. From these results, it is demonstrated that the system states are forced to be bounded closely to the desired trajectories. The tracking errors of the RLSC system can be bounded within a relatively smaller region around zero since the proposed RLSC can alleviate the fluctuations. Figure 19 and Figure 20 depict the front steering wheel angle and the yaw moment control input of this case, separately. As can be seen from these figures, the concerned two inputs can be bounded in reasonable regions. Similar to the results of Case 1, it is observed that the optimized control inputs determined by RLSC can mitigate the overshoots to a smaller region of zero compared to the traditional methods. In order to handle the unknown disturbance and improve system robustness, the traditional SMC may require overestimated control gains, which may result in a relatively large chattering of the tracking errors. Moreover, Figure 21 and Figure 22 demonstrate the scheduling tendency of the control gain $\sigma_{2}$ and the estimated uncertainties in this case. Since the control gains can be adaptively tuned to accommodate the time-varying operating conditions, the proposed RLSC has potential for reducing the errors with respect to yaw rate and sideslip angle tracking. Figure 23 and Figure 24 show the corresponding sliding mode surfaces of yaw rate and sideslip angle, respectively. As can be seen from these figures, without the adaption of the control gains, the resulting sliding surfaces have a large range of variation, which may be caused by the system uncertainties and disturbances. Utilizing the proposed RLSC method with a fuzzy-based estimator to handle the unknown disturbances adaptively, one can obtain smaller and smoother sliding variables, achieving enhanced tracking response in terms of convergence and stability.

In this case, with our newly developed RLSC method, it is concluded that the overshoot and oscillation of the achieved trajectory are significantly mitigated for global dynamical tracing. Therefore, the active disturbance suppression and lateral stabilization control goal is reached more satisfactorily applying our presented RLSC scheme. This verifies the effectiveness and practicability of the super-twisting sliding mode mechanism and fuzzy-based estimator.

## 5. Conclusions

This paper proposes an improved RLSC method to achieve the anti-disturbance lateral motion control of a developed IWMD-MR. This method can be used to precisely regulate the yaw rate and sideslip angle without system information relating to the lumped disturbances/uncertainties and the corresponding derivatives. To this end, a fuzzy-based estimator is incorporated into the construction of the adaptive super-twisting sliding mode mechanism, which can eliminate the dynamic perturbations and time-varying lumped disturbances in an active way. This new scheme ensures that the output states can asymptotically arrive at the finite-time sliding region to achieve enhanced control precision and system robustness. With the aid of the Lyapunov theory, the stability and finite-time convergence are ensured for the resulting lateral motion control system. Real-time simulation experimental results verify that the lateral stabilization control performance of the developed IWMD-MR is improved as compared to the comparative traditional methods.

While the presented method can significantly enhance lateral motion control performance, the maneuvering mode considered in this work is limited to traditional car-like Ackerman mode. For further enhancement of RLSC performance in terms of yaw rate and sideslip angle tracking, future work may include studying the switched control scheme under various operating modes, such as double-Ackerman mode and zero-radius steering mode. How to adjust these available modes online will be our future research directions to further improve the lateral stabilization control performances of the developed IWMD-MR system.

## Notations and Abbreviations

The following key notations and abbreviations are used in this paper.

**Notations/Abbreviations**	**Descriptions**
IWMD-MR	in-wheel-motor-driven mobile robots
RLSC	robust lateral stabilization control
AFSC	active front steering control
DYC	direct yaw control
SMC	sliding mode control
PID	proportional integral derivative
m	total mass
$\upsilon_{x}$	longitudinal speed at the left of gravity
$F_{i}^{x}$, $F_{i}^{y}$	longitudinal and lateral tire forces at *i*th tire
β	sideslip angle
γ	yaw rate
$I_{z}$	inertia moment
$L_{f}$, $L_{r}$	distances from the front and rear axles
$\delta_{f}$, $\delta_{r}$	front and rear steering angles
$M_{\omega }$	yaw moment generated by the traction moment of four wheels
d	track width
$F_{rr}^{x},$$F_{rl}^{x}$, $F_{fr}^{x}$, $F_{fl}^{x}$	lateral forces of the rear-right, rear-left, front-right, and front-left tires
$C_{f}$, $C_{r}$	cornering stiffnesses of the front and rear tires
$\sigma_{1}$, $\sigma_{2}$	adaptive control gains
*α*	fractional order
$\mu_{i=1,2}$	predefined coefficients
$\hat{g}(x|{\hat{\theta }}_{g})$	estimated uncertainties
ρ, ε, δ, $\sigma_{1}^{m}$, η	arbitrary constants
